# *N*-(2-Hy­droxy­benz­yl)adamantan-1-aminium bromide

**DOI:** 10.1107/S1600536811026742

**Published:** 2011-07-09

**Authors:** Tao Rong

**Affiliations:** a Ordered Matter Science Research Center, Southeast UniVersity, Nanjing 210096, People’s Republic of China

## Abstract

There are two independent ion pairs in the asymmetric unit of the title compound, C_17_H_24_NO^+^·Br^−^. In the crystal, the ions are linked by inter­molecular N—H⋯Br and O—H⋯Br hydrogen bonds.

## Related literature

The title compound was studied as part of our work to obtain potential ferroelectric phase-change materials. For general background to ferroelectric organic frameworks, see: Fu *et al.* (2009 ▶); Ye *et al.* (2006 ▶); Zhang *et al.* (2008 ▶, 2010 ▶). For a related structure of the adamantyl ring, see: Cheng *et al.* (2008 ▶).
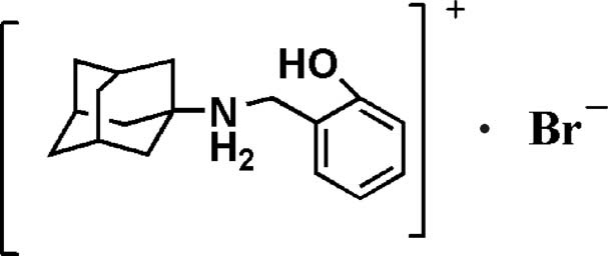


## Experimental

### 

#### Crystal data

C_17_H_24_NO^+^·Br^−^
*M* *_r_* = 338.28
Triclinic, 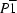

*a* = 10.616 (2) Å
*b* = 12.627 (3) Å
*c* = 12.896 (3) Å
α = 108.46 (3)°
β = 104.69 (3)°
γ = 93.88 (3)°
*V* = 1565.4 (7) Å^3^
*Z* = 4
Mo *K*α radiation
μ = 2.62 mm^−1^
*T* = 293 K
0.20 × 0.20 × 0.20 mm

#### Data collection

Rigaku SCXmini diffractometer
Absorption correction: multi-scan (*CrystalClear*; Rigaku, 2005 ▶) *T*_min_ = 0.596, *T*_max_ = 0.598
16356 measured reflections
7165 independent reflections
5368 reflections with *I* > 2σ(*I*)
*R*_int_ = 0.050

#### Refinement

*R*[*F* ^2^ > 2σ(*F* ^2^)] = 0.045
*wR*(*F* ^2^) = 0.108
*S* = 1.05
7165 reflections
369 parameters
H atoms treated by a mixture of independent and constrained refinement
Δρ_max_ = 0.53 e Å^−3^
Δρ_min_ = −0.55 e Å^−3^

### 

Data collection: *CrystalClear* (Rigaku, 2005 ▶); cell refinement: *CrystalClear*; data reduction: *CrystalClear*; program(s) used to solve structure: *SHELXS97* (Sheldrick, 2008 ▶); program(s) used to refine structure: *SHELXL97* (Sheldrick, 2008 ▶); molecular graphics: *DIAMOND* (Brandenburg & Putz, 2005 ▶); software used to prepare material for publication: *SHELXL97*.

## Supplementary Material

Crystal structure: contains datablock(s) I, global. DOI: 10.1107/S1600536811026742/lx2188sup1.cif

Structure factors: contains datablock(s) I. DOI: 10.1107/S1600536811026742/lx2188Isup2.hkl

Supplementary material file. DOI: 10.1107/S1600536811026742/lx2188Isup3.cml

Additional supplementary materials:  crystallographic information; 3D view; checkCIF report

## Figures and Tables

**Table 1:** Hydrogen-bond geometry (Å, °)

*D*—H⋯*A*	*D*—H	H⋯*A*	*D*⋯*A*	*D*—H⋯*A*
O2—H2*O*⋯Br1^i^	0.82 (4)	2.45 (4)	3.255 (2)	168 (4)
N1—H1*A*⋯Br2^ii^	0.90	2.69	3.527 (3)	155
N1—H1*B*⋯Br2^iii^	0.90	2.45	3.337 (2)	167
N2—H2*A*⋯Br1^iv^	0.90	2.40	3.297 (2)	176
N2—H2*B*⋯Br2^iv^	0.90	2.50	3.377 (2)	165

Symmetry codes: (i) 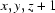; (ii) 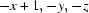; (iii) 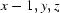; (iv) 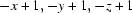.

**Fig. 1.**
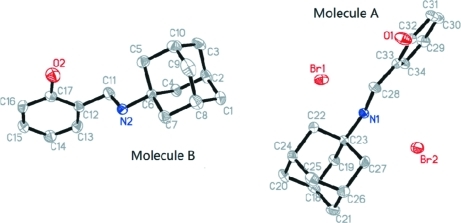
The molecular structure of the title compound, with the atomic numbering scheme. Thermal ellipsoids are shown at the 30% probability level.

**Fig. 2.**
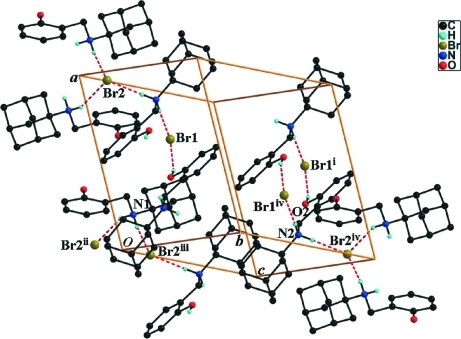
A view of the N—H···Br and O—H···Br interactions (dotted lines) in the crystal structure of the title compound. [Symmetry codes: (i) x, y, z + 1; (ii) - x + 1, - y, - z; (iii) x - 1, y, z; (iv) - x + 1, - y + 1, - z + 1.]

## supplementary crystallographic information

### Comment

The study of ferroelectric materials has received much attention and some materials have predominantly dielectric-ferroelectric performance (Fu *et al.*, 2009; Ye *et al.*, 2006; Zhang *et al.*, 2008, 2010), As a part of our work to obtain potential ferroelectric phase-change materials, we report herein on the crystal structure of title compound. Unluckily, the title compound has no dielectric anomalies in the temperature range 93–53 K, suggesting that it might be only a paraelectric.

The asymmetric unit of the title compoundis is shown in Fig. 1. There are two independent molecules [labelled A & B]. The crystal packing (Fig. 2) is stabilized by weak intermolecular N—H···Br and O—H···Br hydrogen bonds between the *N*-(2-hydroxybenzyl)-1-adamantylammonium cations (Cheng *et al.* 2008) and bromide anions (see; Table 1).

### Experimental

Salicylaldehyde (2.44 g, 20 mmol) and KOH (1.12 g, 20 mmol) were added into a solution of amantadine hydrochloride (3.76 g, 20 mmol) in ethanol. Then a little of anhydrous magnesium sulfate was added into it, after 6 h return the yellow precipitate came out. The yellow solid of amantadine shrink Yang Schiff was obtained by filtration, collection and drying. NaBH_4_ (3.78 g, 10 mmol) was added into a solution of amantadine shrink Yang Schiff (6.38 g, 25 mmol) in anhydrous methanol (120 ml). After 5 h reaction, then the white solid, *N*-(2-hydroxybenzyl)-1-adamantylamine was obtained by reduced pressure distillation, extraction and drying. A solution of hydrobromide (0.8 g, 10 mmol) was added to a solution of *N-*(2-hydroxybenzyl)-1-adamantylamine (2.56 g, 10 mmol) in ethanol (20 ml). Single crystals suitable for *X*-ray diffraction were prepared by slow evaporation of the mixture at room temperature.

### Refinement

The H atoms of OH group were located in a difference density Fourier map and these H atoms were refined freely with an isotropic displacement parameters *U*_iso_ = 1.5*U*_eq_(O). All other H atoms were positioned geometrically and refined using a riding model, with N—H = 0.95Å, C—H = 0.93Å for aryl, 0.98Å for methine and 0.97Å for methylene H atoms, respectively. *U*_iso_(H) = 1.2*U*_eq_(N), and 1.2*U*_eq_(C) for aryl, methine and methylene H atoms.

### Crystal data

**Table:**

C_17_H_24_NO^+^·Br^−^	*Z* = 4
*M**_r_* = 338.28	*F*(000) = 704
Triclinic, *P*1	*D*_x_ = 1.435 Mg m^−^^3^
Hall symbol: -P 1	Mo *K*α radiation, λ = 0.71073 Å
*a* = 10.616 (2) Å	Cell parameters from 7165 reflections
*b* = 12.627 (3) Å	θ = 3.0–27.5°
*c* = 12.896 (3) Å	µ = 2.62 mm^−^^1^
α = 108.46 (3)°	*T* = 293 K
β = 104.69 (3)°	Prism, colourless
γ = 93.88 (3)°	0.20 × 0.20 × 0.20 mm
*V* = 1565.4 (7) Å^3^	

### Data collection

**Table:**

Rigaku SCXmini diffractometer	7165 independent reflections
Radiation source: fine-focus sealed tube	5368 reflections with *I* > 2σ(*I*)
graphite	*R*_int_ = 0.050
Detector resolution: 13.6612 pixels mm^-1^	θ_max_ = 27.5°, θ_min_ = 3.0°
CCD_Profile_fitting scans	*h* = −13→13
Absorption correction: multi-scan (*CrystalClear*; Rigaku, 2005)	*k* = −16→16
*T*_min_ = 0.596, *T*_max_ = 0.598	*l* = −16→16
16356 measured reflections	

### Refinement

**Table:**

Refinement on *F*^2^	Primary atom site location: structure-invariant direct methods
Least-squares matrix: full	Secondary atom site location: difference Fourier map
*R*[*F*^2^ > 2σ(*F*^2^)] = 0.045	Hydrogen site location: difference Fourier map
*wR*(*F*^2^) = 0.108	H atoms treated by a mixture of independent and constrained refinement
*S* = 1.05	*w* = 1/[σ^2^(*F*_o_^2^) + (0.0416*P*)^2^ + 0.2157*P*] where *P* = (*F*_o_^2^ + 2*F*_c_^2^)/3
7165 reflections	(Δ/σ)_max_ = 0.001
369 parameters	Δρ_max_ = 0.53 e Å^−^^3^
0 restraints	Δρ_min_ = −0.55 e Å^−^^3^

### Special details

**Table:**

Geometry. All esds (except the esd in the dihedral angle between two l.s. planes) are estimated using the full covariance matrix. The cell esds are taken into account individually in the estimation of esds in distances, angles and torsion angles; correlations between esds in cell parameters are only used when they are defined by crystal symmetry. An approximate (isotropic) treatment of cell esds is used for estimating esds involving l.s. planes.
Refinement. Refinement of *F*^2^ against ALL reflections. The weighted *R*-factor *wR* and goodness of fit *S* are based on *F*^2^, conventional *R*-factors *R* are based on *F*, with *F* set to zero for negative *F*^2^. The threshold expression of *F*^2^ > σ(*F*^2^) is used only for calculating *R*-factors(gt) *etc*. and is not relevant to the choice of reflections for refinement. *R*-factors based on *F*^2^ are statistically about twice as large as those based on *F*, and *R*- factors based on ALL data will be even larger.

### Fractional atomic coordinates and isotropic or equivalent isotropic displacement parameters (Å^2^)

**Table:**

	*x*	*y*	*z*	*U*_iso_*/*U*_eq_	
Br1	0.63717 (3)	0.35091 (3)	0.25158 (3)	0.04739 (11)	
Br2	0.95230 (3)	0.20644 (3)	0.01025 (3)	0.05202 (12)	
O1	0.3412 (3)	−0.0956 (2)	−0.0911 (2)	0.0595 (7)	
H1O	0.350 (4)	−0.159 (4)	−0.133 (4)	0.089*	
N1	0.2065 (2)	0.07541 (18)	0.07930 (19)	0.0354 (5)	
H1A	0.1895	−0.0006	0.0484	0.043*	
H1B	0.1296	0.1009	0.0614	0.043*	
C1	0.2492 (3)	0.0655 (2)	−0.1044 (2)	0.0393 (7)	
C2	0.1854 (3)	0.1234 (3)	−0.1706 (3)	0.0481 (8)	
H2	0.1656	0.1941	−0.1353	0.058*	
C3	0.1503 (3)	0.0787 (3)	−0.2881 (3)	0.0502 (8)	
H3	0.1069	0.1187	−0.3319	0.060*	
C4	0.1798 (3)	−0.0245 (3)	−0.3394 (3)	0.0473 (7)	
H4	0.1568	−0.0544	−0.4188	0.057*	
C5	0.2426 (3)	−0.0855 (2)	−0.2770 (3)	0.0450 (7)	
H5	0.2613	−0.1562	−0.3135	0.054*	
C6	0.2778 (3)	−0.0407 (2)	−0.1589 (3)	0.0409 (7)	
C7	0.2971 (3)	0.1177 (3)	0.0236 (3)	0.0487 (8)	
H7A	0.3844	0.1005	0.0508	0.058*	
H7B	0.3039	0.1993	0.0455	0.058*	
C8	0.3704 (3)	0.0488 (3)	0.2401 (3)	0.0485 (8)	
H8A	0.4422	0.0723	0.2143	0.058*	
H8B	0.3442	−0.0324	0.2036	0.058*	
C9	0.2549 (3)	0.1080 (2)	0.2089 (2)	0.0329 (6)	
C10	0.4152 (3)	0.0800 (3)	0.3700 (3)	0.0580 (9)	
H10	0.4909	0.0430	0.3915	0.070*	
C11	0.3040 (4)	0.0408 (3)	0.4091 (3)	0.0658 (10)	
H11A	0.3330	0.0588	0.4913	0.079*	
H11B	0.2785	−0.0406	0.3733	0.079*	
C12	0.4553 (3)	0.2068 (3)	0.4270 (3)	0.0555 (9)	
H12A	0.4850	0.2262	0.5093	0.067*	
H12B	0.5276	0.2324	0.4029	0.067*	
C13	0.3391 (3)	0.2646 (3)	0.3947 (3)	0.0498 (8)	
H13	0.3660	0.3467	0.4317	0.060*	
C14	0.1411 (3)	0.0686 (3)	0.2471 (3)	0.0492 (8)	
H14A	0.1140	−0.0125	0.2102	0.059*	
H14B	0.0663	0.1052	0.2256	0.059*	
C15	0.2955 (3)	0.2355 (2)	0.2650 (2)	0.0399 (7)	
H15A	0.2218	0.2731	0.2431	0.048*	
H15B	0.3675	0.2608	0.2404	0.048*	
C16	0.1863 (3)	0.0988 (3)	0.3772 (3)	0.0578 (9)	
H16	0.1140	0.0733	0.4026	0.069*	
C17	0.2266 (3)	0.2272 (3)	0.4337 (3)	0.0583 (9)	
H17A	0.1522	0.2644	0.4126	0.070*	
H17B	0.2540	0.2477	0.5161	0.070*	
O2	0.3888 (2)	0.43144 (18)	1.1055 (2)	0.0513 (6)	
H2O	0.445 (4)	0.412 (3)	1.150 (3)	0.077*	
N2	0.1643 (2)	0.55623 (17)	0.87142 (17)	0.0301 (5)	
H2A	0.2172	0.5849	0.8389	0.036*	
H2B	0.1351	0.6146	0.9147	0.036*	
C18	0.5517 (3)	0.7260 (3)	1.2349 (3)	0.0489 (8)	
H18	0.6185	0.7729	1.2983	0.059*	
C19	0.4831 (3)	0.7692 (2)	1.1562 (3)	0.0467 (7)	
H19	0.5045	0.8449	1.1656	0.056*	
C20	0.3824 (3)	0.6999 (2)	1.0633 (2)	0.0407 (7)	
H20	0.3351	0.7301	1.0112	0.049*	
C21	0.3507 (3)	0.5867 (2)	1.0463 (2)	0.0327 (6)	
C22	0.4228 (3)	0.5436 (2)	1.1257 (2)	0.0348 (6)	
C23	0.5217 (3)	0.6137 (3)	1.2199 (3)	0.0445 (7)	
H23	0.5681	0.5847	1.2733	0.053*	
C24	0.2453 (3)	0.5038 (2)	0.9480 (2)	0.0378 (6)	
H24A	0.2862	0.4469	0.9035	0.045*	
H24B	0.1877	0.4660	0.9775	0.045*	
C25	0.0462 (2)	0.4793 (2)	0.7770 (2)	0.0277 (5)	
C26	0.0911 (3)	0.3764 (2)	0.7046 (2)	0.0327 (6)	
H26A	0.1287	0.3326	0.7510	0.039*	
H26B	0.1580	0.4005	0.6740	0.039*	
C27	−0.0289 (3)	0.3042 (2)	0.6069 (2)	0.0370 (6)	
H27	−0.0019	0.2375	0.5595	0.044*	
C28	−0.1328 (3)	0.2664 (2)	0.6566 (3)	0.0463 (8)	
H28A	−0.2083	0.2190	0.5952	0.056*	
H28B	−0.0962	0.2224	0.7032	0.056*	
C29	−0.0868 (3)	0.3728 (2)	0.5343 (2)	0.0446 (7)	
H29A	−0.1617	0.3265	0.4715	0.053*	
H29B	−0.0212	0.3970	0.5025	0.053*	
C30	−0.1761 (3)	0.3700 (3)	0.7293 (3)	0.0443 (7)	
H30	−0.2430	0.3454	0.7608	0.053*	
C31	−0.2341 (3)	0.4387 (3)	0.6565 (3)	0.0503 (8)	
H31A	−0.2616	0.5046	0.7028	0.060*	
H31B	−0.3108	0.3933	0.5952	0.060*	
C32	−0.1304 (3)	0.4758 (2)	0.6070 (2)	0.0409 (7)	
H32	−0.1676	0.5199	0.5597	0.049*	
C33	−0.0113 (3)	0.5488 (2)	0.7043 (2)	0.0359 (6)	
H33A	0.0547	0.5739	0.6736	0.043*	
H33B	−0.0380	0.6150	0.7509	0.043*	
C34	−0.0571 (3)	0.4427 (2)	0.8272 (2)	0.0390 (7)	
H34A	−0.0838	0.5086	0.8742	0.047*	
H34B	−0.0206	0.3996	0.8748	0.047*	

### Atomic displacement parameters (Å^2^)

**Table:**

	*U*^11^	*U*^22^	*U*^33^	*U*^12^	*U*^13^	*U*^23^
Br1	0.03813 (18)	0.0543 (2)	0.0625 (2)	0.01073 (14)	0.01487 (15)	0.03648 (17)
Br2	0.04467 (19)	0.04178 (18)	0.0533 (2)	0.01298 (14)	0.00594 (16)	−0.00015 (14)
O1	0.0695 (16)	0.0559 (14)	0.0533 (15)	0.0148 (13)	0.0061 (13)	0.0273 (12)
N1	0.0318 (12)	0.0330 (12)	0.0376 (13)	0.0032 (10)	0.0085 (11)	0.0086 (10)
C1	0.0353 (16)	0.0419 (16)	0.0349 (15)	−0.0051 (13)	0.0103 (13)	0.0075 (13)
C2	0.054 (2)	0.0377 (16)	0.054 (2)	0.0096 (14)	0.0210 (17)	0.0133 (15)
C3	0.054 (2)	0.0493 (19)	0.0493 (19)	0.0120 (15)	0.0110 (16)	0.0223 (16)
C4	0.0513 (19)	0.0492 (18)	0.0353 (16)	0.0022 (15)	0.0080 (15)	0.0114 (14)
C5	0.0535 (19)	0.0349 (16)	0.0433 (17)	0.0094 (14)	0.0155 (15)	0.0073 (13)
C6	0.0388 (16)	0.0393 (16)	0.0438 (17)	0.0017 (13)	0.0100 (14)	0.0160 (14)
C7	0.0458 (18)	0.0559 (19)	0.0381 (17)	−0.0088 (15)	0.0135 (15)	0.0102 (14)
C8	0.0531 (19)	0.0467 (18)	0.0486 (19)	0.0197 (15)	0.0150 (16)	0.0179 (15)
C9	0.0308 (14)	0.0308 (14)	0.0327 (15)	0.0004 (11)	0.0089 (12)	0.0061 (11)
C10	0.054 (2)	0.067 (2)	0.058 (2)	0.0211 (18)	0.0085 (18)	0.0310 (19)
C11	0.090 (3)	0.051 (2)	0.051 (2)	−0.0070 (19)	0.009 (2)	0.0233 (17)
C12	0.0389 (18)	0.074 (2)	0.0422 (18)	−0.0123 (16)	−0.0001 (15)	0.0184 (17)
C13	0.058 (2)	0.0369 (16)	0.0394 (17)	−0.0080 (15)	0.0072 (16)	0.0021 (13)
C14	0.0389 (17)	0.0560 (19)	0.0426 (18)	−0.0118 (14)	0.0098 (15)	0.0090 (15)
C15	0.0491 (18)	0.0262 (14)	0.0387 (16)	0.0028 (12)	0.0081 (14)	0.0080 (12)
C16	0.049 (2)	0.074 (2)	0.0435 (19)	−0.0169 (18)	0.0161 (16)	0.0153 (17)
C17	0.056 (2)	0.072 (2)	0.0354 (17)	0.0099 (18)	0.0124 (16)	0.0042 (16)
O2	0.0446 (13)	0.0427 (12)	0.0608 (15)	0.0043 (10)	−0.0049 (11)	0.0266 (11)
N2	0.0297 (12)	0.0304 (11)	0.0270 (11)	0.0019 (9)	0.0058 (10)	0.0081 (9)
C18	0.0383 (17)	0.0465 (18)	0.0435 (18)	0.0033 (14)	−0.0022 (14)	0.0029 (14)
C19	0.0441 (18)	0.0358 (16)	0.0521 (19)	0.0048 (13)	0.0064 (15)	0.0107 (14)
C20	0.0386 (16)	0.0420 (16)	0.0378 (16)	0.0079 (13)	0.0034 (13)	0.0145 (13)
C21	0.0283 (14)	0.0378 (15)	0.0315 (14)	0.0071 (11)	0.0099 (12)	0.0098 (12)
C22	0.0283 (14)	0.0390 (15)	0.0370 (15)	0.0078 (12)	0.0084 (12)	0.0134 (12)
C23	0.0360 (16)	0.0545 (19)	0.0365 (16)	0.0092 (14)	−0.0002 (13)	0.0153 (14)
C24	0.0391 (16)	0.0381 (15)	0.0337 (15)	0.0065 (12)	0.0023 (13)	0.0155 (12)
C25	0.0280 (13)	0.0272 (13)	0.0237 (13)	0.0020 (10)	0.0045 (11)	0.0060 (10)
C26	0.0335 (14)	0.0334 (14)	0.0297 (14)	0.0055 (11)	0.0092 (12)	0.0089 (11)
C27	0.0419 (16)	0.0272 (13)	0.0329 (15)	0.0037 (12)	0.0085 (13)	0.0008 (11)
C28	0.0456 (18)	0.0334 (15)	0.0494 (18)	−0.0055 (13)	0.0039 (15)	0.0103 (14)
C29	0.0491 (18)	0.0455 (17)	0.0279 (15)	−0.0019 (14)	0.0009 (14)	0.0077 (13)
C30	0.0342 (16)	0.0470 (17)	0.0484 (18)	−0.0052 (13)	0.0149 (14)	0.0120 (14)
C31	0.0301 (16)	0.0461 (18)	0.058 (2)	0.0037 (13)	0.0014 (15)	0.0053 (15)
C32	0.0381 (16)	0.0380 (16)	0.0375 (16)	0.0040 (13)	−0.0043 (13)	0.0132 (13)
C33	0.0385 (16)	0.0310 (14)	0.0345 (15)	0.0032 (12)	0.0045 (13)	0.0117 (12)
C34	0.0385 (16)	0.0449 (16)	0.0345 (15)	0.0019 (13)	0.0178 (13)	0.0101 (13)

### Geometric parameters (Å, °)

**Table:**

O1—C6	1.358 (4)	O2—C22	1.361 (3)
O1—H1O	0.84 (4)	O2—H2O	0.82 (4)
N1—C7	1.500 (4)	N2—C24	1.487 (3)
N1—C9	1.525 (3)	N2—C25	1.520 (3)
N1—H1A	0.9000	N2—H2A	0.9000
N1—H1B	0.9000	N2—H2B	0.9000
C1—C2	1.375 (4)	C18—C19	1.372 (4)
C1—C6	1.396 (4)	C18—C23	1.372 (4)
C1—C7	1.503 (4)	C18—H18	0.9300
C2—C3	1.378 (4)	C19—C20	1.380 (4)
C2—H2	0.9300	C19—H19	0.9300
C3—C4	1.360 (4)	C20—C21	1.380 (4)
C3—H3	0.9300	C20—H20	0.9300
C4—C5	1.370 (4)	C21—C22	1.394 (4)
C4—H4	0.9300	C21—C24	1.495 (4)
C5—C6	1.384 (4)	C22—C23	1.381 (4)
C5—H5	0.9300	C23—H23	0.9300
C7—H7A	0.9700	C24—H24A	0.9700
C7—H7B	0.9700	C24—H24B	0.9700
C8—C9	1.512 (4)	C25—C34	1.522 (4)
C8—C10	1.530 (5)	C25—C33	1.524 (3)
C8—H8A	0.9700	C25—C26	1.526 (4)
C8—H8B	0.9700	C26—C27	1.531 (4)
C9—C15	1.521 (3)	C26—H26A	0.9700
C9—C14	1.525 (4)	C26—H26B	0.9700
C10—C11	1.508 (5)	C27—C29	1.516 (4)
C10—C12	1.513 (5)	C27—C28	1.529 (4)
C10—H10	0.9800	C27—H27	0.9800
C11—C16	1.524 (5)	C28—C30	1.524 (4)
C11—H11A	0.9700	C28—H28A	0.9700
C11—H11B	0.9700	C28—H28B	0.9700
C12—C13	1.510 (5)	C29—C32	1.521 (4)
C12—H12A	0.9700	C29—H29A	0.9700
C12—H12B	0.9700	C29—H29B	0.9700
C13—C17	1.511 (5)	C30—C31	1.519 (4)
C13—C15	1.530 (4)	C30—C34	1.527 (4)
C13—H13	0.9800	C30—H30	0.9800
C14—C16	1.533 (4)	C31—C32	1.521 (4)
C14—H14A	0.9700	C31—H31A	0.9700
C14—H14B	0.9700	C31—H31B	0.9700
C15—H15A	0.9700	C32—C33	1.526 (4)
C15—H15B	0.9700	C32—H32	0.9800
C16—C17	1.530 (5)	C33—H33A	0.9700
C16—H16	0.9800	C33—H33B	0.9700
C17—H17A	0.9700	C34—H34A	0.9700
C17—H17B	0.9700	C34—H34B	0.9700
			
C6—O1—H1O	109 (3)	C22—O2—H2O	108 (3)
C7—N1—C9	116.9 (2)	C24—N2—C25	116.5 (2)
C7—N1—H1A	108.1	C24—N2—H2A	108.2
C9—N1—H1A	108.1	C25—N2—H2A	108.2
C7—N1—H1B	108.1	C24—N2—H2B	108.2
C9—N1—H1B	108.1	C25—N2—H2B	108.2
H1A—N1—H1B	107.3	H2A—N2—H2B	107.3
C2—C1—C6	118.5 (3)	C19—C18—C23	120.0 (3)
C2—C1—C7	121.4 (3)	C19—C18—H18	120.0
C6—C1—C7	119.9 (3)	C23—C18—H18	120.0
C1—C2—C3	121.3 (3)	C18—C19—C20	119.9 (3)
C1—C2—H2	119.3	C18—C19—H19	120.1
C3—C2—H2	119.3	C20—C19—H19	120.1
C4—C3—C2	119.2 (3)	C19—C20—C21	121.1 (3)
C4—C3—H3	120.4	C19—C20—H20	119.5
C2—C3—H3	120.4	C21—C20—H20	119.5
C3—C4—C5	121.5 (3)	C20—C21—C22	118.4 (3)
C3—C4—H4	119.2	C20—C21—C24	125.6 (2)
C5—C4—H4	119.2	C22—C21—C24	116.1 (2)
C4—C5—C6	119.3 (3)	O2—C22—C23	123.2 (3)
C4—C5—H5	120.4	O2—C22—C21	116.5 (2)
C6—C5—H5	120.4	C23—C22—C21	120.3 (3)
O1—C6—C5	122.7 (3)	C18—C23—C22	120.3 (3)
O1—C6—C1	117.1 (3)	C18—C23—H23	119.8
C5—C6—C1	120.1 (3)	C22—C23—H23	119.8
N1—C7—C1	112.0 (2)	N2—C24—C21	113.7 (2)
N1—C7—H7A	109.2	N2—C24—H24A	108.8
C1—C7—H7A	109.2	C21—C24—H24A	108.8
N1—C7—H7B	109.2	N2—C24—H24B	108.8
C1—C7—H7B	109.2	C21—C24—H24B	108.8
H7A—C7—H7B	107.9	H24A—C24—H24B	107.7
C9—C8—C10	108.5 (3)	N2—C25—C34	110.6 (2)
C9—C8—H8A	110.0	N2—C25—C33	106.36 (19)
C10—C8—H8A	110.0	C34—C25—C33	109.5 (2)
C9—C8—H8B	110.0	N2—C25—C26	109.8 (2)
C10—C8—H8B	110.0	C34—C25—C26	110.6 (2)
H8A—C8—H8B	108.4	C33—C25—C26	110.0 (2)
C8—C9—C15	110.3 (2)	C25—C26—C27	108.3 (2)
C8—C9—N1	109.2 (2)	C25—C26—H26A	110.0
C15—C9—N1	110.2 (2)	C27—C26—H26A	110.0
C8—C9—C14	109.8 (2)	C25—C26—H26B	110.0
C15—C9—C14	109.8 (2)	C27—C26—H26B	110.0
N1—C9—C14	107.4 (2)	H26A—C26—H26B	108.4
C11—C10—C12	109.4 (3)	C29—C27—C28	109.5 (2)
C11—C10—C8	109.6 (3)	C29—C27—C26	109.8 (2)
C12—C10—C8	110.0 (3)	C28—C27—C26	109.3 (2)
C11—C10—H10	109.3	C29—C27—H27	109.4
C12—C10—H10	109.3	C28—C27—H27	109.4
C8—C10—H10	109.3	C26—C27—H27	109.4
C10—C11—C16	109.7 (3)	C30—C28—C27	109.5 (2)
C10—C11—H11A	109.7	C30—C28—H28A	109.8
C16—C11—H11A	109.7	C27—C28—H28A	109.8
C10—C11—H11B	109.7	C30—C28—H28B	109.8
C16—C11—H11B	109.7	C27—C28—H28B	109.8
H11A—C11—H11B	108.2	H28A—C28—H28B	108.2
C13—C12—C10	109.6 (3)	C27—C29—C32	109.7 (2)
C13—C12—H12A	109.7	C27—C29—H29A	109.7
C10—C12—H12A	109.7	C32—C29—H29A	109.7
C13—C12—H12B	109.7	C27—C29—H29B	109.7
C10—C12—H12B	109.7	C32—C29—H29B	109.7
H12A—C12—H12B	108.2	H29A—C29—H29B	108.2
C12—C13—C17	110.3 (3)	C31—C30—C28	109.9 (3)
C12—C13—C15	109.1 (3)	C31—C30—C34	109.6 (2)
C17—C13—C15	110.2 (3)	C28—C30—C34	109.3 (2)
C12—C13—H13	109.1	C31—C30—H30	109.3
C17—C13—H13	109.1	C28—C30—H30	109.3
C15—C13—H13	109.1	C34—C30—H30	109.3
C9—C14—C16	109.1 (2)	C30—C31—C32	109.2 (2)
C9—C14—H14A	109.9	C30—C31—H31A	109.8
C16—C14—H14A	109.9	C32—C31—H31A	109.8
C9—C14—H14B	109.9	C30—C31—H31B	109.8
C16—C14—H14B	109.9	C32—C31—H31B	109.8
H14A—C14—H14B	108.3	H31A—C31—H31B	108.3
C9—C15—C13	108.5 (2)	C29—C32—C31	110.1 (2)
C9—C15—H15A	110.0	C29—C32—C33	109.2 (2)
C13—C15—H15A	110.0	C31—C32—C33	109.3 (2)
C9—C15—H15B	110.0	C29—C32—H32	109.4
C13—C15—H15B	110.0	C31—C32—H32	109.4
H15A—C15—H15B	108.4	C33—C32—H32	109.4
C11—C16—C17	109.7 (3)	C25—C33—C32	109.0 (2)
C11—C16—C14	109.0 (3)	C25—C33—H33A	109.9
C17—C16—C14	109.0 (3)	C32—C33—H33A	109.9
C11—C16—H16	109.7	C25—C33—H33B	109.9
C17—C16—H16	109.7	C32—C33—H33B	109.9
C14—C16—H16	109.7	H33A—C33—H33B	108.3
C13—C17—C16	108.9 (3)	C25—C34—C30	108.7 (2)
C13—C17—H17A	109.9	C25—C34—H34A	110.0
C16—C17—H17A	109.9	C30—C34—H34A	110.0
C13—C17—H17B	109.9	C25—C34—H34B	110.0
C16—C17—H17B	109.9	C30—C34—H34B	110.0
H17A—C17—H17B	108.3	H34A—C34—H34B	108.3
			
C6—C1—C2—C3	−0.2 (4)	C23—C18—C19—C20	1.2 (5)
C7—C1—C2—C3	175.1 (3)	C18—C19—C20—C21	−1.4 (5)
C1—C2—C3—C4	−0.2 (5)	C19—C20—C21—C22	0.2 (4)
C2—C3—C4—C5	0.6 (5)	C19—C20—C21—C24	−179.0 (3)
C3—C4—C5—C6	−0.5 (5)	C20—C21—C22—O2	−179.7 (3)
C4—C5—C6—O1	−179.4 (3)	C24—C21—C22—O2	−0.4 (4)
C4—C5—C6—C1	0.1 (4)	C20—C21—C22—C23	1.1 (4)
C2—C1—C6—O1	179.8 (3)	C24—C21—C22—C23	−179.6 (3)
C7—C1—C6—O1	4.4 (4)	C19—C18—C23—C22	0.1 (5)
C2—C1—C6—C5	0.3 (4)	O2—C22—C23—C18	179.6 (3)
C7—C1—C6—C5	−175.2 (3)	C21—C22—C23—C18	−1.3 (4)
C9—N1—C7—C1	170.8 (2)	C25—N2—C24—C21	−173.7 (2)
C2—C1—C7—N1	101.4 (3)	C20—C21—C24—N2	−5.7 (4)
C6—C1—C7—N1	−83.3 (3)	C22—C21—C24—N2	175.1 (2)
C10—C8—C9—C15	60.2 (3)	C24—N2—C25—C34	66.7 (3)
C10—C8—C9—N1	−178.6 (2)	C24—N2—C25—C33	−174.5 (2)
C10—C8—C9—C14	−61.0 (3)	C24—N2—C25—C26	−55.5 (3)
C7—N1—C9—C8	−67.9 (3)	N2—C25—C26—C27	−177.1 (2)
C7—N1—C9—C15	53.4 (3)	C34—C25—C26—C27	60.7 (3)
C7—N1—C9—C14	173.1 (3)	C33—C25—C26—C27	−60.4 (3)
C9—C8—C10—C11	61.0 (3)	C25—C26—C27—C29	60.0 (3)
C9—C8—C10—C12	−59.3 (4)	C25—C26—C27—C28	−60.0 (3)
C12—C10—C11—C16	59.9 (4)	C29—C27—C28—C30	−59.4 (3)
C8—C10—C11—C16	−60.8 (4)	C26—C27—C28—C30	60.8 (3)
C11—C10—C12—C13	−60.2 (4)	C28—C27—C29—C32	59.4 (3)
C8—C10—C12—C13	60.3 (4)	C26—C27—C29—C32	−60.5 (3)
C10—C12—C13—C17	60.4 (3)	C27—C28—C30—C31	59.7 (3)
C10—C12—C13—C15	−60.7 (3)	C27—C28—C30—C34	−60.6 (3)
C8—C9—C14—C16	60.8 (3)	C28—C30—C31—C32	−59.5 (3)
C15—C9—C14—C16	−60.7 (3)	C34—C30—C31—C32	60.7 (3)
N1—C9—C14—C16	179.4 (3)	C27—C29—C32—C31	−59.8 (3)
C8—C9—C15—C13	−61.1 (3)	C27—C29—C32—C33	60.2 (3)
N1—C9—C15—C13	178.2 (2)	C30—C31—C32—C29	59.5 (3)
C14—C9—C15—C13	60.0 (3)	C30—C31—C32—C33	−60.5 (3)
C12—C13—C15—C9	60.6 (3)	N2—C25—C33—C32	179.6 (2)
C17—C13—C15—C9	−60.6 (3)	C34—C25—C33—C32	−60.9 (3)
C10—C11—C16—C17	−59.4 (4)	C26—C25—C33—C32	60.8 (3)
C10—C11—C16—C14	59.8 (4)	C29—C32—C33—C25	−60.0 (3)
C9—C14—C16—C11	−59.4 (3)	C31—C32—C33—C25	60.5 (3)
C9—C14—C16—C17	60.3 (4)	N2—C25—C34—C30	177.5 (2)
C12—C13—C17—C16	−59.4 (3)	C33—C25—C34—C30	60.7 (3)
C15—C13—C17—C16	61.1 (4)	C26—C25—C34—C30	−60.7 (3)
C11—C16—C17—C13	58.7 (4)	C31—C30—C34—C25	−60.6 (3)
C14—C16—C17—C13	−60.6 (4)	C28—C30—C34—C25	59.9 (3)

### Hydrogen-bond geometry (Å, °)

**Table:**

*D*—H···*A*	*D*—H	H···*A*	*D*···*A*	*D*—H···*A*
O2—H2O···Br1^i^	0.82 (4)	2.45 (4)	3.255 (2)	168 (4)
N1—H1A···Br2^ii^	0.90	2.69	3.527 (3)	155
N1—H1B···Br2^iii^	0.90	2.45	3.337 (2)	167
N2—H2A···Br1^iv^	0.90	2.40	3.297 (2)	176
N2—H2B···Br2^iv^	0.90	2.50	3.377 (2)	165

Symmetry codes: (i) *x*, *y*, *z*+1; (ii) −*x*+1, −*y*, −*z*; (iii) *x*−1, *y*, *z*; (iv) −*x*+1, −*y*+1, −*z*+1.

## References

[bb1] Brandenburg, K. & Putz, H. (2005). *DIAMOND* Crystal Impact GbR, Bonn, Germany.

[bb2] Cheng, L., Xu, X. & Xu, Y. (2008). *Acta Cryst.* E**64**, m82.

[bb3] Fu, D. W., Ge, J. Z., Dai, J., Ye, H. Y. & Qu, Z. R. (2009). *Inorg. Chem. Commun.* **12**, 994–997.

[bb4] Rigaku (2005). *CrystalClear* Rigaku Corporation, Tokyo, Japan.

[bb5] Sheldrick, G. M. (2008). *Acta Cryst.* A**64**, 112–122. 10.1107/S0108767307043930 18156677

[bb6] Ye, Q., Song, Y. M., Wang, G. X., Chen, K. & Fu, D. W. (2006). *J. Am. Chem. Soc.* **128**, 6554–6555. 10.1021/ja060856p 16704244

[bb7] Zhang, W., Xiong, R. G. & Huang, S. P. D. (2008). *J. Am. Chem. Soc.* **130**, 10468–10469. 10.1021/ja803021v 18636707

[bb8] Zhang, W., Ye, H. Y., Cai, H. L., Ge, J. Z. & Xiong, R. G. (2010). *J. Am. Chem. Soc.* **132**, 7300–7302. 10.1021/ja102573h 20459097

